# Evaluation of Microtensile Bond Strength of a 3D‐Printed Hybrid Material Under Various Adhesive Strategies

**DOI:** 10.1002/cre2.70363

**Published:** 2026-04-28

**Authors:** Patricia Freire‐Nieto, Lissethe Peñate‐González, Luis Jané‐Chimeno, Anaïs Ramírez‐Sebastià, Miguel Roig‐Cayón, Marta Vallés‐Rodríguez

**Affiliations:** ^1^ Department of Restorative Dentistry, Faculty of Dentistry Universitat Internacional de Catalunya Barcelona Spain; ^2^ Department of Endodontics, Faculty of Dentistry Universitat Internacional de Catalunya Barcelona Spain

**Keywords:** adhesion, definitive crown, microtensile bond strength, printed hybrid material

## Abstract

**Objectives:**

Printed dental materials have emerged to overcome the limitations associated with the subtractive CAD/CAM technologies. The aim of the present study was to evaluate the optimal adhesive strategy for Permanent Crown Resin, a printed hybrid material.

**Material and Methods:**

Eighteen specimens of Permanent Crown Resin were divided into three groups of six blocks each, measuring 30 × 30 × 10 mm. A specific surface pretreatment was performed, involving two groups undergoing hydrofluoric acid etching (60 s) and sandblasting (20 s), whereas the third group remained devoid of any pretreatment. Comprehensive surface characterization was achieved using a scanning electron microscope (SEM) at magnifications of 500× and 1000×. Each of the main groups was further divided into three subgroups, each containing two blocks. The paired blocks within each subgroup were then cemented to each other using different cementation protocols: a dual‐cure self‐adhesive resin cement RelyX^TM^ Unicem 2, a dual‐cure adhesive ExciTE® F DSC, followed by dual‐cure resin cement Variolink® Esthetic DC, and a light‐cure universal adhesive Scotchbond^TM^ Universal in combination with preheated composite Filtek^TM^ Supreme XTE. Subsequently, the specimens were sectioned, and 20 sound micro‐bars were obtained from each specimen (*n* = 180). Each micro‐bar was subjected to microtensile bond strength (μTBS) testing at a crosshead speed of 0.5 mm/min.

**Results:**

SEM analysis revealed a smooth surface in the control group, whereas the HF group showed numerous micropores. The sandblasting group showed the roughest surface. Statistical analysis was performed using two‐way ANOVA, and a *p*‐value of ≤ 0.05 was considered statistically significant. Regarding the μTBS test, sandblasting pretreatment combined with RelyX^TM^ Unicem 2 demonstrated higher bond strength than the other groups (60.23 MPa). The predominant mode of failure across all groups was adhesive.

**Conclusions:**

Within the limitations of this study, sandblasting in conjunction with RelyX^TM^ Unicem 2 provided the highest bond strength for Permanent Crown Resin.

## 1 Introduction

Computer‐aided design (CAD) and/or computer‐aided manufacturing (CAM) have gained widespread acceptance. These technologies are commonly used in the production of dental prosthetics within machining centers, where excess material is removed from a block to achieve the desired shape. This process is known as subtractive manufacturing (SM) (Galante et al. 2019; Van Noort 2012). CAD/CAM technologies have significantly enhanced the field of dentistry by enabling the production of reliable restorations with precise dimensions while reducing the manufacturing time (Dehurtevent et al. 2017; Bindl and MÖRMANN 2005). However, these processes have limitations, including raw material wastage, challenges in recycling excess material, substantial wear of milling tools, and the formation of microscopic cracks (Wang et al. 2008).

In contemporary dentistry, a novel technological advancement known as additive manufacturing (AM), also called 3D printing, has emerged. This process enables the construction of dental prosthetics by incrementally adding materials layer by layer, guided by a computerized 3D model (Huang et al. 2013). Notably, the layered composition of the final restoration mitigates the accumulation of tooling stresses associated with milling procedures (Methani et al. 2020; Huang 2003). In prosthetic dentistry, AM has gained considerable commercial recognition for the fabrication of metal crowns, copings, and resin‐based components (Zeng et al. 2015). However, the use of AM for fabricating ceramic prostheses remains an ongoing area of development (Methani et al. 2020; Ebert et al. 2009).

Although CAD/CAM systems mainly use ceramic materials due to their esthetic qualities, surface finish, and long‐term durability, these materials are characterized by low fracture toughness and high brittleness (Ruse and Sadoun 2014; Şişmanoğlu et al. 2020; Vichi et al. 2014; Della Bona et al. 2014a). A novel category of CAD/CAM materials referred to as resin–matrix ceramics (RMCs) has been introduced to address these shortcomings. Based on their microstructure, these hybrid materials are categorized into two groups: resin‐based CAD/CAM composites and polymer‐infiltrated ceramic network (PICN).

Resin‐based CAD/CAM composites are composite–ceramic restorative materials that combine the advantages of a highly cross‐linked resin matrix and ceramic (Şişmanoğlu et al. 2020; Schepke et al. 2016; Bonfante et al. 2015). Brands producing this type of material include Cerasmart^TM^ (GC Corporation), Lava^TM^ Ultimate (3 M ESPE), BRILLIANT Crios (Coltene), SHOFU Block HC (Shofu Inc.), or Grandio® blocs (Voco GmbH). PICN materials, exemplified by VITA ENAMIC® (VITA Zahnfabrik; Bad Säckingen), are interpenetrating phase composite materials formed by the infiltration of 14% resin into an 86% ceramic network (Şişmanoğlu et al. 2020; Coldea et al. 2013a; Della Bona et al. 2014b). Thus, these materials have a hybrid surface that can be treated as both indirect composite and ceramic materials.

Hybrid materials combine the advantages of ceramics and composites, thereby demonstrating superior mechanical properties (Mine et al. 2019; Spitznagel et al. 2014). Thanks to their enhanced fracture toughness and reduced brittleness, they have emerged as a viable alternative to ceramics (Şişmanoğlu et al. 2020; Coldea et al. 2013b).

In this context, CAD/CAM hybrid materials served as precursors to the development of hybrid printed materials. In late 2020, a groundbreaking printed hybrid material entered the market. BEGO Bremer Goldschlägerei Wilh. Herbst GmbH & Co. KG introduced a definitive printed hybrid resin (ceramic‐filled resin) designed for 3D printing of permanent restorations, including single crowns, inlays, onlays, and veneers for anterior and posterior areas. This resin is commercialized by BEGO under the name of VarseoSmile Crown ^plus^ and Permanent Crown Resin by Formlabs Inc.

CAD/CAM and printed hybrid materials are primarily used for indirect adhesive restorations. The adhesive protocol of CAD/CAM hybrid materials depends upon their compositions. For resin‐based CAD/CAM composites, the gold standard protocol involves a two‐step process comprising sandblasting with aluminum oxide as a surface pretreatment, followed by the application of an adhesive. It is noteworthy that universal adhesives show promising potential as effective material primers in this context. Conversely, the use of hydrofluoric acid and a silane coupling agent appears to have a less favorable impact on resin‐based CAD/CAM composites. In contrast, for PICN materials, the preferred surface pretreatment involves hydrofluoric acid etching, followed by silane application (Emsermann et al. 2019).

Given that Permanent Crown Resin is indicated for definitive restorations, factors such as shade matching, optical integration, and digital workflow are relevant for clinical application (Ozturk et al. 2025). Moreover, the adhesive bonding strategies investigated in this study are consistent with the principles of minimally invasive dentistry, which emphasize preservation of healthy tooth structure and conservative restorative interventions. Accurate diagnosis and careful treatment planning are key components of this approach, and adjunctive tools such as laser fluorescence could complement conventional diagnostic methods to enhance reliability while supporting minimally invasive procedures (Sinanoglu et al. 2014).

It is important to note that although the manufacturer suggests adhesive luting to bond Permanent Crown Resin to the tooth structure, it does not specify the optimal adhesive strategy. Therefore, the purpose of this study was to assess various adhesive strategies for bonding Permanent Crown Resin.

## 2 Materials and Methods

The present study investigated the adhesive protocol for printed hybrid materials, with a specific focus on Permanent Crown Resin.

The sample size was determined using Granmo calculator software (IMIM) for a two‐sided test with an alpha risk of 0.05 and a beta risk of 0.2. To detect a statistically minimum difference of 5 units between any pair of groups, 21 subjects were required for each group. A standard deviation of 4 was assumed, and no drop‐out rate was anticipated.

### 2.1 Specimen Preparation

**Figure 1:**
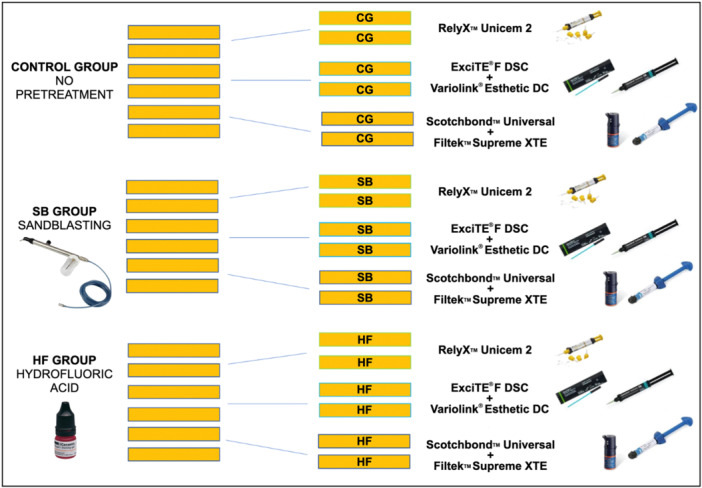
Study groups.

Eighteen blocks of Permanent Crown Resin, each measuring 30 mm × 30 mm × 10 mm, were printed. Both sides of these printed blocks were subjected to polishing to standardize the surfaces using 600‐ and 1000‐grit silicon carbide abrasive papers (Hitech Europe MP Series, Tecmet 2000). Subsequently, the specimens were immersed in an ultrasonic bath (XUBA3, Grant; UK) for 5 min to eliminate the impurities. The 18 blocks were divided into three groups of six blocks each (Figure 1). Different surface pretreatments were applied to the bonding surfaces of each randomly selected printed block, as follows: a control group with no surface pretreatment (CG), etching with hydrofluoric acid (HF), and sandblasting with 50‐μm aluminum oxide (Al_2_O_3_) particles (SB).

The specimens were exposed to 5% hydrofluoric acid (IPS Ceramic Etching Gel, Ivoclar Vivadent; Schaan, Liechtenstein) for 1 min, rinsed using an air–water spray for 1 min, cleaned in an ultrasonic bath filled with distilled water for 5 min, and then air‐dried. In this study, a silane coupling agent was not applied after hydrofluoric acid etching. This methodological choice was made to specifically evaluate the bond strength of resin cements to the untreated printed hybrid material and to isolate the effects of surface pretreatment alone.

Sandblasting was conducted using a sandblaster machine (Airsonic® Mini‐Sandblaster, Hager Werken) at a distance of approximately 5 mm from the surface of the Permanent Crown Resin block. The specimens were sandblasted with 50‐μm Al_2_O_3_ particles for 20 s, and a pressure of 2 bar was maintained for air abrasion. Afterward, all sandblasted specimens were cleaned in an ultrasonic bath filled with distilled water for 5 min and then air‐dried.

Subsequently, the specimens were analyzed with a scanning electron microscope (SEM) (JEOL JSM 5410, JEOL Ltd.) operating at 10 kV with magnifications of 500× and 1000×. Before observation, the specimens were coated with a gold layer using an Agar sputter coater (AGB7340, Agar Scientific Ltd.).

Following the SEM analysis, each of the three groups was further divided into three subgroups, each containing two blocks. These pairs of blocks within each subgroups were then cemented directly to each other using different types of cementation (Figure 1).

In the first subgroup, each specimen was cemented with the dual self‐adhesive resin cement RelyX^TM^ Unicem 2 (3 M ESPE). For the second subgroup, the dual‐cure adhesive ExciTE® F DSC (Ivoclar Vivadent; Schaan, Liechtenstein) was directly applied onto the surface of the two specimens for 20 s, followed by air‐drying. Subsequently, each specimen was cemented with dual‐cure resin cement Variolink® Esthetic DC neutral (Ivoclar Vivadent; Schaan, Liechtenstein). In the third subgroup, the light‐cure universal adhesive Scotchbond^TM^ Universal (3 M ESPE) was directly applied to the surface of the two specimens for 20 s, followed by air‐drying. Subsequently, each specimen was cemented with the Filtek^TM^ Supreme XTE composite (3 M ESPE). The composite was preheated to approximately 55°C for 10 min before application with a specific device (ENA Heat Composite Heating Conditioner, Micerium S.p.A.). All cementation processes were conducted under a 1 kg weight for 5 min, followed by polymerization using a light‐emitting diode curing device (Bluephase Style i20, Ivoclar Vivadent; Schaan, Liechtenstein) for 20 s, in accordance with the manufacturer's instructions. The materials, their compositions, and the manufacturer of each material are listed in Table 1.

**Table 1:** Overview of the characteristics of the tested materials.

Trade name and manufacturer	Type of material	Composition
Permanent Crown Resin (Formlabs Inc.)	Ceramic‐filled hybrid material	4'‐isopropylidiphenol, ethoxylated, and 2‐methylprop‐2enoic acid. Silanized dental glass, methyl benzoylformate, and diphenyl (2,4,6‐trimethylbenzoyl) phosphine oxide 30–50 wt. % of inorganic fillers (particle size 0.7 μm)
Airsonic Aluminum‐Oxyd Pulver (Hager Werken)	Surface conditioner	50‐μm aluminum oxide (Al_2_O_3_) particles
IPS Ceramic Etching Gel (Ivoclar Vivadent)	Ceramic conditioner	5% Hydrofluoric acid
RelyX^TM^ Unicem 2 (3 M ESPE)	Dual self‐adhesive resin cement	Catalyst paste: glass powder, surface‐modified with 2‐propenoic acid, 2 methyl‐.3‐(trimethoxysilyl)propyl ester, bulk material, substituted dimethacrylate, 1,12‐dodecane dimethycrylate, 2,4,6(1H,3H,5H)‐pyrimidinetrione, 5‐phenyl‐1‐ (phenylmethyl)‐, calcium salt (2:1), silane‐treated silica, sodium p‐toluenesulfinate, 2‐propenoic acid, 2‐methyl‐, [(3‐ methoxypropyl)imino]di‐2,1‐ethanediyl ester, calcium hydroxide, methacrylated amine, and titanium dioxide. Base paste: glass powder, surface‐modified with 2‐propenoic acid, 2 methyl‐3‐(trimethoxysilyl)propyl ester and phenyltrimethoxy silane, bulk material, 2‐propenoic acid, 2‐methyl‐, 1,1'‐[1‐(hydroxymethyl)‐1,2‐ethanediyl] ester, reaction products with 2‐hydroxy‐1,3‐ propanediyl dimethacrylate and phosphorus oxide, TEG‐DMA, silane‐treated silica, oxide glass chemicals (non‐fibrous), sodium persulfate, tert‐butyl peroxy‐3,5,5‐ trimethylhexanoate, acetic acid, copper(2+) salt, and monohydrate
ExciTE® F DSC (Ivoclar Vivadent)	Dual‐cure adhesive	HEMA, dimethacrylate, phosphonic acid acrylate, highly dispersed silicone dioxide, initiators, stabilizers, and potassium fluoride in an alcohol solution
Variolink® Esthetic DC (Ivoclar Vivadent)	Dual‐cure resin cement	Ytterbium trifluoride 20 to < 25%, urethane dimethacrylate 5 to < 10%, glycerin‐1,3‐dimethacrylate 3%–7%, 1,10‐decandiol dimethacrylate 3%–7%, spheroid mixed oxide (67 wt %/38 vol %), initiators, stabilizers, and pigments
Scotchbond^TM^ Universal (3 M ESPE)	Light‐cure universal adhesive	10‐MDP Phosphate Monomer, dimethacrylate resins, HEMA, VitrebondTM Copolymer, filler, ethanol, water, initiators, and silane
Filtek^TM^ Supreme XTE (3 M ESPE)	Light‐cure nanofilled composite	Matrix: Bis‐phenol A diglycidylmethacrylate (Bis‐GMA), triethylene glycol dimethacrylate (TEGDMA), urethane dimethacrylate (UDMA), and bis‐phenol A polyethylene glycol diether dimethacrylate Filler: silica nanofillers (5–75 nm) and zirconia/silica nanoclusters (0.6–1.4 μm)

**Figure 2:**
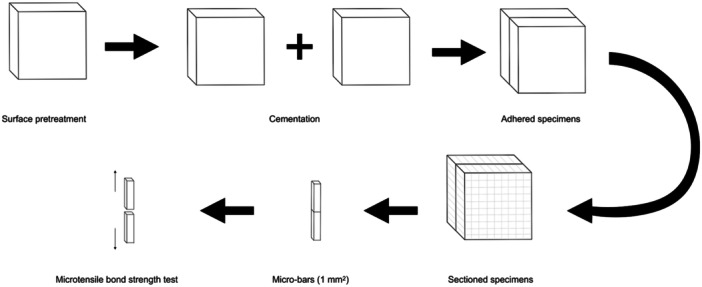
Schematic representation of specimen preparation for the microtensile bond strength test.

The bilayered specimens were sectioned into micro‐bars (1 ± 0.2 mm^2^ in cross section) using a diamond‐coated disk under water cooling (Isomet 1000, Buehler; Illinois, USA). Twenty sound micro‐bars were obtained from each specimen (*n* = 180) and stored in distilled water for 24 h at 37°C (Figure 2). Before carrying out the microtensile bond strength (μTBS) test, the specimens were subjected to aging in a thermocycling machine (9106A11B, PolyScience) for 20,000 cycles at temperatures ranging from 5°C to 55°C, with a dwell time of 30 s and a transfer time of 5 s. The micro‐bars were affixed to a custom‐made attachment unit and subjected to tensile force using a universal testing machine at a crosshead speed of 0.5 mm/min (Z005 ProLine, ZwickRoell GmbH & Co.). The fractured surface of each micro‐bar was examined under a stereomicroscope (SteREO Discovery.V8, Carl Zeiss) at 2.5× magnification to assess the failure mode, which was categorized as adhesive (fracture line through the resin adhesive), cohesive (fracture line through the hybrid printed material), or mixed (a combination of the above).

The results were analyzed using the statistical software Statgraphics Centurion XVI (StatPoint Technologies Inc.). The Kolmogorov–Smirnov test was applied to assess normality. A two‐way ANOVA test was performed, and a *p*‐value of ≤ 0.05 was considered statistically significant. Additionally, Fisher's exact test was conducted to analyze the fracture type.

## 3 Results

Statistically significant differences were observed in the μTBS test between the three surface pretreatments (*p* = 0.0001) and the three types of cementation (*p* < 0.0109).

Table 2 shows the results of the μTBS test, measured in megapascals (MPa), for the Permanent Crown Resin material pretreated and cemented using various strategies. The mean and standard deviation of the μTBS results were calculated for each group.

**Table 2:** Means ± standard deviation (SD) in Mpa of μTBS.

		**Cementation**		
		**Relyx** ^**TM**^ **Unicem 2**	**Scotchbond** ^**TM**^ **Universal** + **Filtek** ^**TM**^ **Supreme XTE**	**Excite**® **F DSC** + **Variolink**® **Esthetic DC**
Surface pretreatment	No pretreatment	56.86 (15.5)^Aa^	51.58 (20.7)^Aa^	33.82 (11.2)^Ab^
	Hydrofluoric acid	37.27 (13.1)^Ba^	44.18 (17.1)^Aa^	35.29 (21.7)^Aa^
	Sandblasting	60.23 (7.9)^Aa^	48.60 (15.4)^Ab^	57.64 (20.2)^Bab^

Different lowercase letters indicate statistically significant differences between cementation groups (*p* ≤ 0.05). Different uppercase letters indicate statistically significant differences between surface pretreatment groups (*p* ≤ 0.05).

### 3.1 Surface Pretreatment Effect

When evaluating the effect of the pretreatment, regardless of the type of cementation used, sandblasting pretreatment demonstrated significantly superior results compared to the HF pretreatment and no pretreatment (*p* = 0.0001). Where no pretreatment was applied, cementation with RelyX^TM^ Unicem 2 or Scotchbond^TM^ Universal in combination with preheated composite showed significantly superior results to the combination of ExciTE® F DSC and Variolink® Esthetic DC (*p* = 0.0001). Conversely, when HF pretreatment was applied, no statistically significant differences were found among the three types of cementation (*p* = 0.2608), resulting in low µTBS values. However, with sandblasting as the chosen pretreatment method, cementation with RelyX^TM^ Unicem 2 significantly outperformed the combination of Scotchbond^TM^ Universal and a preheated composite, achieving the highest µTBS values (mean 60.23 MPa). The optimal combination appears to be the use of sandblasting in conjunction with RelyX^TM^ Unicem 2, as it showed the highest resistance, measured in MPa, during the microtensile bond strength test.

### 3.2 Cementation Effect

When evaluating the influence of cementation, regardless of the pretreatment method, the use of RelyX^TM^ Unicem 2 and Scotchbond^TM^ Universal combined with a preheated composite demonstrated a significant advantage over the combination of ExciTE® F DSC and Variolink® Esthetic DC (*p* = 0.0109). Upon analyzing each type of cementation separately, it became evident that, in the case of ExciTE® F DSC combined with the Variolink® Esthetic DC group, the µTBS values showed a significant increase when the sandblasting surface pretreatment was used (*p* = 0.002). The RelyX^TM^ Unicem 2 group showed high µTBS values when subjected to sandblasting or when no surface pretreatment was applied, both of which were significantly superior to cases when HF pretreatment was used (*p* = 0.0001). This suggests that HF pretreatment has a detrimental effect on the achieved adhesion in the case of cementation with RelyX^TM^ Unicem 2. However, the adhesion values after cementation with Scotchbond^TM^ Universal and preheated composite appeared to be unaffected by the analyzed surface pretreatments.

### 3.3 Failure Mode Analysis

Fisher's exact test was used to assess the relationship between the failure mode and both the surface pretreatment and cementation type. This study revealed no statistically significant relationship between the failure mode and the cementation type (*p* = 0.064) or the surface pretreatment performed (*p* = 0.233).

**Figure 3:**
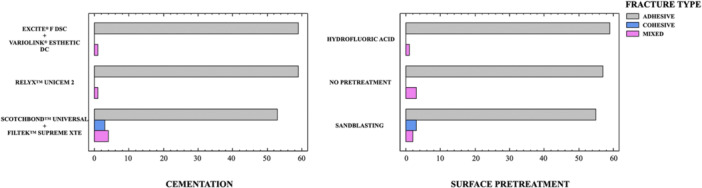
Failure types according to cementation and pretreatment.

**Figure 4:**
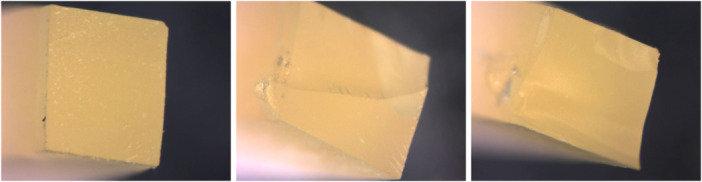
Representative images obtained by a stereomicroscope (2.5× magnification) of the fractured surfaces of the specimens. From left to right: adhesive failure, mixed failure, and cohesive failure.

Figure 3 shows the percentages of fracture types within each group. The failure mode analysis indicated a higher prevalence of adhesive fractures overall (95%), whereas mixed fractures (3.3%) and cohesive fractures (1.7%) were less common. More specifically, the percentages of adhesive fractures, categorized by the type of surface pretreatment, were 32.7% in the HF group, 31.7% in the no pretreatment group, and 30.6% in the sandblasting group. Regarding the type of cementation, adhesive fractures were 32.7% in the ExciTE® F DSC group, 32.7% in the RelyX^TM^ Unicem 2 group, and 29.4% in the Scotchbond^TM^ Universal group. Cohesive failures were only observed in the group where sandblasting and Scotchbond^TM^ Universal were combined (1.7%), whereas mixed failures (3.3%) were evenly distributed among the groups. As a result, adhesive failures were the prevailing fracture type in all specimen groups, with cohesive and mixed failure types being exceptionally rare (see Figure 4).

### 3.4 Surface Topography

**Figure 5:**
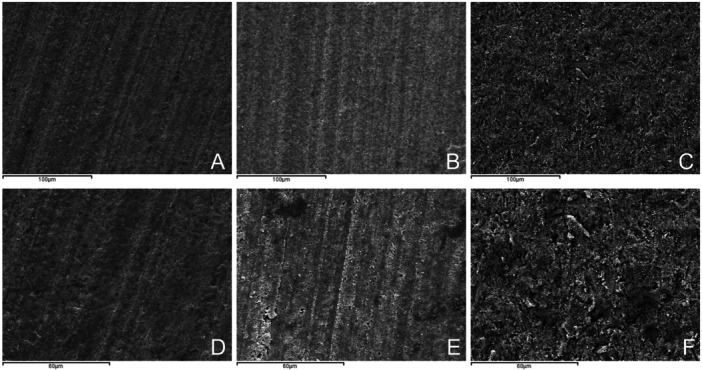
Representative scanning electron microscope (SEM) images of the surface of the specimens after pretreatments at 500× magnification. (A) No surface pretreatment. (B) Etching with hydrofluoric acid (HF). (C,) Sandblasting with 50‐μm aluminum oxide (Al_2_O_3_) particles. Representative SEM images of the surface of the specimens after pretreatments at 1000× magnification. (D) No surface pretreatment. (E) Etching with hydrofluoric acid (HF). (F) Sandblasting with 50‐μm aluminum oxide (Al_2_O_3_) particles.

The specimens were subjected to examination with a SEM. Representative micrographs of the hybrid surfaces following the various surface pretreatments are shown in Figure 5. SEM analysis revealed distinct surface topographies for the restorative material depending upon the type of surface pretreatment used. The control group (no pretreatment) showed a uniform and smooth surface appearance. In contrast, the HF group showed a surface marked by micropores, stemming from the partial dissolution of the glassy phase due to the effect of hydrofluoric acid. The sandblasting group showed a rougher surface compared to the other groups, characterized by embedded blasting particles of varying sizes and shapes along with the presence of some grooves.

## 4 Discussion

The aim of this study was to determine the optimal strategy for bonding the printed hybrid material Permanent Crown Resin. The outcomes confirmed the initial research hypothesis. The first hypothesis posited that the type of surface pretreatment would influence the μTBS results, which was supported by these findings. The second hypothesis, which postulated that the type of cementation would yield variations in μTBS results, was also confirmed.

The μTBS test was used to evaluate the bonding performance of cementation strategies. This test is more appropriate than the shear bond strength test (SBS), which often leads to cohesive fractures distant from the resin interface, introducing inaccuracies in assessment (Sano et al. 1994; Keshvad and Hakimaneh 2018).

Laboratory studies frequently use artificial aging protocols to replicate clinical conditions, including thermocycling, storage in aqueous solutions, and fatigue techniques, each characterized by distinct parameters (Papadopoulos et al. 2021). In the present study, the specimens were stored in distilled water for 24 h at 37°C, followed by aging in a thermocycling machine for 20,000 cycles, ranging between 5°C and 55°C. Thermocycling is commonly used to simulate the effects of aging in the oral environment; however, its direct equivalence to a specific clinical time frame remains controversial (Gale and Darvell 1999). Previous research demonstrated a decrease in bond strength after 6‐month aging, underscoring the significant impact of storage duration (aging) on bond strength (Papadopoulos et al. 2021; Lise et al. 2017).

Comparisons with other studies are limited, as this is among the first studies to observe the adhesion on this printed hybrid material using the μTBS test. However, we can draw parallels with similar CAD/CAM hybrid materials. According to the manufacturer's specifications, Permanent Crown Resin is a heterogeneous material primarily composed of resin, with ceramic particles representing 30% to 50% of the total weight, comprising 0.7 µm inorganic fillers of silanized dental glass. Previous research has demonstrated that both ceramic thickness and material type can influence the micromechanical properties of light‐cured adhesive bonding agents. Notably, although ceramic type may affect adhesive hardness, the curing performance and mechanical behavior are primarily determined by the adhesive itself, and less influenced by ceramic thickness or curing time. Therefore, differences in filler content, particle size, and polymer network structure may significantly affect the adhesive performance, light transmission, and polymerization behavior of resin cements (Öztürk et al. 2014).

Various resin‐based CAD/CAM blocks are available in the market, each with distinct compositions. SHOFU Block HC contains SiO_2_ and ZrO_2_ filler particles (61% weight). In contrast, Lava^TM^ Ultimate contains a higher percentage of SiO_2_ and ZrO_2_ (80% weight) nanofillers as well as larger aggregated clusters. BRILLIANT Crios consists of barium glass and SiO_2_ fillers (71% weight), whereas Cerasmart^TM^ incorporates silica and barium glass (71% weight). Notably, VITA ENAMIC® differs significantly from the others, as it is a polymer‐infiltrated ceramic composed of two different networks, predominantly ceramic (86% weight) with a polymer component (14% weight). It is important to note that VITA ENAMIC® shows a unique structure characterized by two continuous interconnected networks, in stark contrast to the composition of Permanent Crown Resin (Spitznagel et al. 2018). Furthermore, SHOFU Block HC and Lava^TM^ Ultimate contain zirconia particles in their compositions, which are absent in Permanent Crown Resin. Therefore, among the available CAD/CAM blocks, Cerasmart^TM^ and BRILLIANT Crios appear to be the closest chemical counterparts to Permanent Crown Resin, despite their higher percentage of ceramic particles.

The use of aluminum oxide particle‐based sandblasting has gained widespread support as an effective approach for enhancing surface characteristics, such as roughness, energy, and wettability (Keshvad and Hakimaneh 2018). Elsaka, in 2014, reported that increased surface roughness could lead to a larger surface area, potentially improving the bond between CAD/CAM materials and luting agents (Elsaka 2014). Studies on Cerasmart^TM^ and VITA ENAMIC® concluded that the creation of a microretentive surface through sandblasting or HF etching, followed by silanization, led to a significant increase in the µTBS values (Lise et al. 2017). Similarly, Graf et al. found that airborne‐particle abrasion showed higher adhesion values and avoided possible retention loss on the surfaces of VITA ENAMIC® and VarseoSmile Crown ^plus^ (Graf et al. 2022). The effects of different sandblasting powder particles were also explored, and it was found that the size of particles did not significantly influence μTBS results (Tekçe et al. 2019). Therefore, it appears that air abrasion as a surface pretreatment is imperative for establishing a reliable bond with resin‐based CAD/CAM composite blocks.

In this investigation, focusing solely on the effect of the surface pretreatment, it was observed that sandblasting improved the adhesion between the material and the luting agent significantly. The superior performance of sandblasting combined with the resin cement RelyX^TM^ Unicem 2 is consistent with previous findings on CAD/CAM Resin–matrix ceramics, which reported higher microtensile bond strength values for resin cements after airborne‐particle abrasion than after other surface treatments. Notably, the effect of surface treatment on μTBS was more pronounced than the effects of ceramic type or resin cement, emphasizing the critical role of mechanical surface pretreatment in optimizing bond strength (Bayazıt 2019).

In contrast, the use of the hydrofluoric acid had a notably detrimental impact. It is well established that acid‐based surface treatments could significantly influence bond strength outcomes. For example, Öztürk et al. demonstrated in an in vitro study that reducing phosphoric acid etching time on dentin from 15 to 3 s significantly increased the microtensile bond strength of a bulk‐fill composite using universal adhesives. Although the substrate and type of acid differ from the present study, the findings highlight the importance of etching parameters, which may partially explain the variable bond strength observed after hydrofluoric acid etching of Permanent Crown Resin (Öztürk et al. 2025). However, it should be noted that silane was not applied after HF etching in this study, which may have had the potential to enhance adhesion values. This aligns with a study by Park and Choi, which evaluated the influence of sandblasting and HF etching on the bond strength of Lava^TM^ Ultimate material, thus providing further corroboration of our results (Park and Choi 2016). In a later study, Reymus et al. tested specimens pretreated with or without air abrasion, followed by either a silane primer or a universal adhesive. Notably, air abrasion significantly increased tensile bond strength values, and specimens pretreated with a universal adhesive showed significantly higher tensile bond strength values than those pretreated with a silane primer (Reymus et al. 2019).

In the current study, no statistically significant differences between sandblasting and HF etching were found in the group where Scotchbond^TM^ Universal was used. This finding might be attributed to its composition, which contains 10‐MDP and silane, potentially augmenting adhesion values after HF application. It is noteworthy that no chemical pretreatment involving silane application was performed in our study; however, the use of a silane primer might affect the adhesion results.

According to SEM images, Papadopoulos et al. reported that mechanical pretreatments, such as sandblasting and CoJet^TM^, resulted in a roughened surface with irregular craters of varying shapes and sizes. Hydrofluoric acid etching treatment with silane had a different effect, revealing exposed glass particles with small pits and micropores (Papadopoulos et al. 2020). In this research, the SEM images of the sandblasting group confirmed these findings, showing a rougher surface, characterized by blasting particles embedded along the surface, alongside some grooves. The HF‐treated groups showed a surface with micropores. However, this etching did not appear to substantially enhance the resistance in the μTBS values. This outcome can be attributed to the fact that Permanent Crown Resin contains a limited amount of ceramic in its composition.

This study has several limitations. First, as an in vitro study, the results may not fully reflect clinical performance due to the complex oral environment. Second, a silane coupling agent was not applied after hydrofluoric acid etching, which could potentially affect the bond strength of some resin cements. Third, only three surface pretreatments and three cementation protocols were evaluated, and variability among the micro‐bars could have influenced the microtensile bond strength measurements. These limitations should be considered when interpreting the results, and further studies are recommended to confirm and expand these findings.

An additional limitation of this study is that the bonding protocol involved cementing two resin blocks to each other rather than bonding the printed material to tooth substrates such as enamel or dentin. Despite this, evaluation of resin‐to‐resin interfaces provides valuable insight into the intrinsic adhesive performance of different surface pretreatments and cementation protocols, reducing the variability introduced by the natural tooth structure. Therefore, the results can be considered indicative of potential clinical performance, although direct extrapolation to clinical scenarios should be interpreted with caution. Future studies involving enamel and dentin substrates are recommended to confirm these findings and assess the effectiveness of the tested bonding strategies under conditions closer to the clinical situation.

## 5 Conclusion

Within the limitations of the study, the following findings were obtained:

1. The optimal bonding strategy for the hybrid printed material Permanent Crown Resin involves the use of sandblasting as a surface pretreatment in conjunction with the resin cement RelyX^TM^ Unicem 2.
2. Regardless of surface modification or cementation protocols, adhesive failure at the interface between samples was the most prevalent mode of failure observed across all groups.

## Author Contributions

**Patricia Freire‐Nieto** and **Marta Vallés‐Rodríguez:** conceptualization. **Patricia Freire‐Nieto, Lissethe Peñate‐González,** and **Marta Vallés‐Rodríguez:** methodology. **Patricia Freire‐Nieto, Lissethe Peñate‐González, Luis Jané‐Chimeno,** and **Anaïs Ramírez‐Sebastià:** investigation. **Patricia Freire‐Nieto** and **Marta Vallés‐Rodríguez:** formal analysis. **Patricia Freire‐Nieto:** data curation. **Patricia Freire‐Nieto:** writing – original draft. **Lissethe Peñate‐González, Luis Jané‐Chimeno, Anaïs Ramírez‐Sebastià, Miguel Roig‐Cayón,** and **Marta Vallés‐Rodríguez:** writing – review and editing. **Miguel Roig‐Cayón** and **Marta Vallés‐Rodríguez:** supervision.

## Funding

The authors have nothing to report.

## Conflicts of Interest

The authors declare no conflicts of interest.

## Data Availability

The data that support the findings of this study are available from the corresponding author upon reasonable request.
